# Augmenting Microarray Data with Literature-Based Knowledge to Enhance Gene Regulatory Network Inference

**DOI:** 10.1371/journal.pcbi.1003666

**Published:** 2014-06-12

**Authors:** Guocai Chen, Michael J. Cairelli, Halil Kilicoglu, Dongwook Shin, Thomas C. Rindflesch

**Affiliations:** Lister Hill National Center for Biomedical Communications, National Library of Medicine, Bethesda, Maryland, United States of America; University of Chicago, United States of America

## Abstract

Gene regulatory networks are a crucial aspect of systems biology in describing molecular mechanisms of the cell. Various computational models rely on random gene selection to infer such networks from microarray data. While incorporation of prior knowledge into data analysis has been deemed important, in practice, it has generally been limited to referencing genes in probe sets and using curated knowledge bases. We investigate the impact of augmenting microarray data with semantic relations automatically extracted from the literature, with the view that relations encoding gene/protein interactions eliminate the need for random selection of components in non-exhaustive approaches, producing a more accurate model of cellular behavior. A genetic algorithm is then used to optimize the strength of interactions using microarray data and an artificial neural network fitness function. The result is a directed and weighted network providing the individual contribution of each gene to its target. For testing, we used invasive ductile carcinoma of the breast to query the literature and a microarray set containing gene expression changes in these cells over several time points. Our model demonstrates significantly better fitness than the state-of-the-art model, which relies on an initial random selection of genes. Comparison to the component pathways of the KEGG Pathways in Cancer map reveals that the resulting networks contain both known and novel relationships. The *p53* pathway results were manually validated in the literature. 60% of non-KEGG relationships were supported (74% for highly weighted interactions). The method was then applied to yeast data and our model again outperformed the comparison model. Our results demonstrate the advantage of combining gene interactions extracted from the literature in the form of semantic relations with microarray analysis in generating contribution-weighted gene regulatory networks. This methodology can make a significant contribution to understanding the complex interactions involved in cellular behavior and molecular physiology.

## Author Summary

We have developed a methodology that combines standard computational analysis of gene expression data with knowledge in the literature to identify pathways of gene and protein interactions. We extract the knowledge from PubMed citations using a tool (SemRep) that identifies specific relationships between genes or proteins. We string together networks of individual interactions that are found within citations that refer to the target pathways. Upon this skeleton of interactions, we calculate the weight of the interaction with the gene expression data captured over multiple time points using state-of-the-art analysis algorithms. Not surprisingly, this approach of combining prior knowledge into the analysis process significantly improves the performance of the analysis. This work is most significant as an example of how the wealth of textual data related to gene interactions can be incorporated into computational analysis, not solely to identify this type of pathway (a gene regulatory network) but for any type of similar biological problem.

## Introduction

Gene regulatory networks (GRNs) are DNA-encoded regulatory subsystems in the genome that coordinate input from activator and repressor transcription factors to control various biological functions, including development, cell differentiation, and response to environmental cues. They provide a systems level illustration of physiological function and are composed of modules at varying hierarchical levels [1]. A GRN provides the pathways of gene interactions within the context of location and time [2]. For instance, when a receptor on a particular cell receives a signal and initiates the activation of a transcription factor, the transcription factor increases the expression of the target gene, which in turn alters the production and activation of other pathway components in a cascading manner, changing the behavior of the cell.

The development of microarray technology allowed the discovery of gene regulation to move from individual interactions to thousands of interactions in parallel [3]. However, system-level techniques like microarrays produce large datasets, requiring efficient computational methods to identify significant changes in gene expression and their correlation. One area of systems biology where these computational methods are increasingly applied is for the inference of GRNs [4]–[9]. Although microarray databanks contain a wealth of data in support of the elucidation of GRNs, mammalian datasets are often limited by low or nonexistent replication and too few time points to allow for reliable results. There have been suggestions (e.g. Sîrbu et al. [10]) that the wealth of biological knowledge on possible interactions available in the literature coupled with the limits on available microarray data warrant an attempt to implement an integration of the two to improve reliability of GRN inferencing results.

We propose utilizing knowledge extracted from publications as a network of interactions and then applying microarray data to provide a measure of the quantitative effect of each of the individual interaction components. The qualitative knowledge at the core of our approach is provided by SemRep, a natural language processing system that extracts textual meaning from the biomedical literature in the form of semantic relations called *predications* (subject-relation-object triples). These predications mostly represent mammalian and specifically human interactions, therefore we use quantitative data provided by analysis of publicly available human breast cancer microarray datasets using a genetic algorithm. We compare the fitness of our model to that of a high-performing model from the literature to determine the performance of our technique as well as another model based on time-delay correlation. We also compare our results to KEGG pathways and find not only included interactions but also interactions not included but validated in the literature. We then modified the components of our method to be compatible with yeast data and again compared with the state-of-the-art model. Our method provides a novel approach to enhancing microarray data analysis with knowledge from the literature, and is the first such approach to incorporate semantic relations. Combining microarray data with semantic relations provides a more accurate model of gene interactions directing the behavior of cells, and consequently an enriched understanding of molecular physiology. This supports the discovery of disease mechanisms, which can then be exploited for diagnostic and therapeutic development in medicine.

### Related work

Previous computational models used to reconstruct gene regulatory networks from microarray data have employed techniques such as Hidden Markov, Bayesian network, and stochastic differential equation models [11]–[15]. In a Hidden Markov Model, the real states of genes are treated as hidden variables, and the gene expression values are observed. This permits the states of genes at a given time *t* to be considered as depending only on the previous time point *t-1*. A Bayesian network is a representation of a joint probability distribution. When applying Bayesian networks to genetic regulatory systems, nodes are identified with genes and their expression levels, edges indicate interactions between genes, and conditional distributions describe these interactions. In the stochastic differential equation, the change of expression of a given target gene over two adjacent time points is equal to the accumulation of the weighted results of the sigmoid function of its regulator genes plus a random error. These computational models all reflect one or more aspects of the nature of genes and gene regulatory networks, but computational complexity limits the dimensionality of the modeled networks, and sensitivity to noise in gene expression measurement largely reduces their accuracy.

Genetic algorithms (GA) [16] have also been widely used for the inference of gene regulatory networks [17]–[20]. Genetic algorithms are inspired by Darwin's theory of evolution. Within this methodology, a population of candidate solutions to a problem is created and then evolves over a specified number of generations using phenomena such as cross-over (swapping components from other candidates) and mutation (internal, random changes). The best candidates are propagated through with incremental changes in the overall structure and the final generation becomes the solution. At each generation, fitness functions are used to determine the fittest candidates. A genetic algorithm is a stochastic algorithm and therefore it is highly likely to find global optima and can easily escape local maxima. Since the genetic algorithm execution technique is not dependent on the error surface, it is capable of solving multi-dimensional, non-differential, non-continuous, and even non-parametrical problems, which is the nature of gene expression data. Keedwell et al. used small random gene subsets evaluated by an artificial neural network (ANN) that is optimized by gradient descent to form the population of the genetic algorithm [21]; Liu and Wu used a differential equation to model the GRNs and genetic programming for optimization [22]; Sîrbu et al. compared various evolutionary algorithms for GRN inferencing and found that Keedwell et al.'s method performs the best overall [10], prompting us to use their method for the basis of our model. They also note that using literature-derived knowledge offers the potential for significant improvement over techniques that probe component genes randomly, and our use of such knowledge forms the basis of our deviation from the Keedwell et al.'s model.

With the rapid rate of growth in the biology literature, text mining is increasingly seen as indispensable in managing and discovering new biological knowledge [23]. An active area of research in biological text mining has been extraction of interactions between biomolecular entities (genes, proteins, etc.) from the research literature. Many systems, adopting various representational means (binary interactions, events, etc.) and using a variety of rule-based and machine learning-based techniques, have been proposed for this task. Early systems that focused only on co-occurrence of entities were soon replaced by systems that relied on shallow parsing and hand-crafted syntactic rules to extract binary interactions [24]–[26, among others]. These methods generally provided high precision at the expense of lower recall, in contrast to co-occurrence based methods. More recently, dependency parsing has become the predominant syntactic tool in extracting biological relations, as evidenced by the BioNLP Shared Task competitions [27]. These competitions have also signaled the increasing focus on events as the representational means for biological relations. Most commonly, dependency relations have formed the basis for syntactic features (shortest paths, dependency n-grams, etc.) for machine learning methods, along with lexical (tokens, n-grams, part-of-speech tags, etc.) and semantic (entity types, hypernyms, etc.) features. Best machine learning approaches have included pipeline models based on support vector machines [28]–[29] as well as model combination techniques [30] and joint inference [31]. Some rule-based systems have reported competitive results in this task, as well [32]. Recently, coreference resolution has also been beneficially integrated into several event extraction systems [33]–[34].

As these text mining methods mature, they are increasingly applied to practical needs of biologists, in tasks such as database curation and pathway generation. For example, two tasks in the recent BioNLP 2013 Shared Task competition [27] (Pathway Curation [35] and Gene Regulation Network in Bacteria [36]) investigated the feasibility of automatically constructing such networks from the literature alone. Given a set of relevant biomolecular entities, the former focused on extracting pathway-relevant events (e.g. gene expression, regulation, binding, regulation), while the latter focused on constructing a gene regulation network for the model bacterium *Bacillus subtilis* involving these entities. For the latter task, participating groups could either directly construct a regulation network for the provided entities or extract the interactions from which such a network could be derived using a predefined algorithm that the organizers provided. While the results were encouraging, these tasks remain challenging as evidenced by the limited participation and the fact that both tasks presupposed that the entities involved were already known, thus, addressing only a fraction of the problem of network construction from the literature. From an opposing viewpoint, Miwa et al. [37] focused on linking interactions in biological pathways to supporting evidence from the literature; however, their work does not address the task of pathway construction.

There have been several attempts at improving gene regulatory network modeling by incorporating existing knowledge. For example, Steele et al. used the correlation between different gene concept profiles to calculate the probability of edges in GRNs modeled by Bayesian networks [38]. These profiles are determined by the occurrence of terms in the literature, using the Unified Medical Language System (UMLS) for normalization. Gutierrez-Rios et al. used regulatory interactions described in RegulonDB, a database of the regulatory network of *Escherichia coli* K-12, to establish the network of causal relationships to evaluate the congruence between the literature and whole-genome expression profiles [39]. Additionally, the literature contains examples of efforts to combine literature-derived networks and microarray analysis in contexts other than GRN inferencing. Duarte et al. reconstructed the human metabolomic network depending largely on a manual literature review combined with knowledge extracted from genomic databases and use gene expression analysis to fill in the gap for a subset of network components [40]. Ashley et al. used text mining to derive biological pathways from literature relevant to in-stent restenosis and analyzed which of these were most relevant to the expression profiles identified by microarray analysis of tissue samples from patients with this condition [41]. All of these techniques either use human review to identify asserted interactions or automated approaches to infer them based on co-occurrence of terms. Our approach combines automation, allowing for an exponentially greater survey of the literature, with the identification of assertions in the text by SemRep, moving beyond mere term co-occurrence and increasing the validity of the extracted relations.

Several books, book chapters, and journal reviews are available detailing the pros and cons of various modeling approaches for GRN inferencing [42]–[47]. In addition to a description of various approaches, Karlebach and Shamir [45] also provide a comparative summary of the relative advantages of different types of models for various features. They align GRN models along a spectrum from logical models (e.g. Boolean networks) to continuous models (e.g. linear differential equations) to single-molecule level models (e.g. stochastic simulation models). They identify the logical end of the spectrum as having a decreased detail, less faithfulness to biological reality, lower data quantity needs, and reduced ability to model dynamics, while having greater model size, computational speed, and inferencing ability. Models at the single-molecule level are positioned at the other end of the spectrum, with a higher level of detail, increased faithfulness to biological reality, greater data quantity needs, and increased ability to model dynamics and decreased model size, computational speed, and inferencing ability. Continuous models are positioned in the middle of the spectrum, with moderate levels of the assessed model's characteristics. Although not providing a complete picture of the comparative characteristics of GRN modeling techniques, they give an easily accessible summarization.

### Background

#### SemRep

The idea that the literature holds a wealth of data that can be systematically used in the development of hypotheses has been a point of discussion in biology for over a decade [48]. The challenge is facilitating the extraction of relevant information from the literature. SemRep [49] addresses this challenge by extracting salient content from titles and abstracts of MEDLINE citations in the form of semantic predications, thereby facilitating large-scale analysis of biomedical knowledge. A semantic predication is a subject-predicate-object triple, whose elements are largely derived from ontological knowledge in the UMLS [50]. The subject and object arguments correspond to concepts in the UMLS Metathesaurus and the predicates to relations in an extended version of UMLS Semantic Network. For example, SemRep extracts the predication in (2) from the sentence in (1):

1. In addition to the upregulation of death receptors, *p53* induced the pro-apoptotic *Bcl-2* family members *Bik* and *Bak* and downregulated the anti-apoptotic *Bcl-xL* protein. (PMID: 11313989)
2. *p53-STIMULATES-Bik*

SemRep relies on the UMLS SPECIALIST Lexicon [51], MedPost part-of-speech tagger [52], and a partial syntactic parser (“chunker”) for syntactic analysis. Noun phrases identified by the parser are mapped to UMLS Metathesaurus concepts by the MetaMap program [53]. With respect to gene/protein terms, ABGene [54] and EntrezGene [55] serve as supplementary resources to MetaMap and the UMLS Metathesaurus, respectively. To identify predicate mentions in text, SemRep uses *indicator rules* that map lexical/syntactic constructions, such as verbs, nominalizations, and modifier-head structures, to UMLS Semantic Network relations. For example, one of those rules stipulates that the verb *induce* may indicate the relation STIMULATES, and this rule licenses mapping *induced* in the example above to this relation. The subject and object arguments (p53 and Bik, respectively) are recognized using MetaMap and ABGene and are then normalized to official EntrezGene symbols. Several studies have evaluated predications extracted by SemRep. For example, Ahlers et al. reported 73% precision and 55% recall in pharmacogenomics relations [56]. Another evaluation focused on a specific linguistic structure (nominalizations) and reported 75% precision and 57% recall [57]. The entire MEDLINE database has been preprocessed with SemRep for efficient access, resulting in the SemMedDB database, which contains more than 57 million predications extracted from 21 million citations, as of June 30, 2012 [58]. By normalizing free text to semantic predications, SemRep provides the ability to represent biomedical content as a network of relations. In our model, the nodes of the network represent genes and the edges represent interactions (INTERACTS_WITH, INHIBITS, and STIMULATES) between the genes.

### Microarray data

The breast cancer microarray data used in our experiments comes from NCBI's Gene expression omnibus (GEO: http://www.ncbi.nlm.nih.gov/geo/) [59]. GEO provides free access to raw and processed microarray and sequencing data submitted by researchers based on their published work. For the yeast study, we used the cdc15 time course data from the Yeast Cell Cycle Analysis Project website (http://genome-www.stanford.edu/cellcycle/) originally used in [60].

### KEGG

For assessment of our methodology, we use the Kyoto Encyclopedia of Genes and Genomes (KEGG: http://www.genome.jp/kegg/) [61], which provides gold standard sets of molecular pathways. KEGG pathways are manually curated networks based on a review of protein-gene and protein-protein interactions described in the literature. It is worth noting that KEGG pathway maps are an abbreviated representation of known interactions, focusing on those considered to be best supported by evidence and most relevant. As a consequence, the interactions in a KEGG pathway form a subset of those in the literature, and it is necessary to assess separately the validity of interactions not in the KEGG pathway. A KEGG pathway can be accessed as a downloadable kgml file or an online map, which have slight differences regarding which genes are included and which interactions are specified. To mediate these differences we use both formats for our assessment and consider whether genes and interactions are included in either version.

## Methods

**Figure 1:**
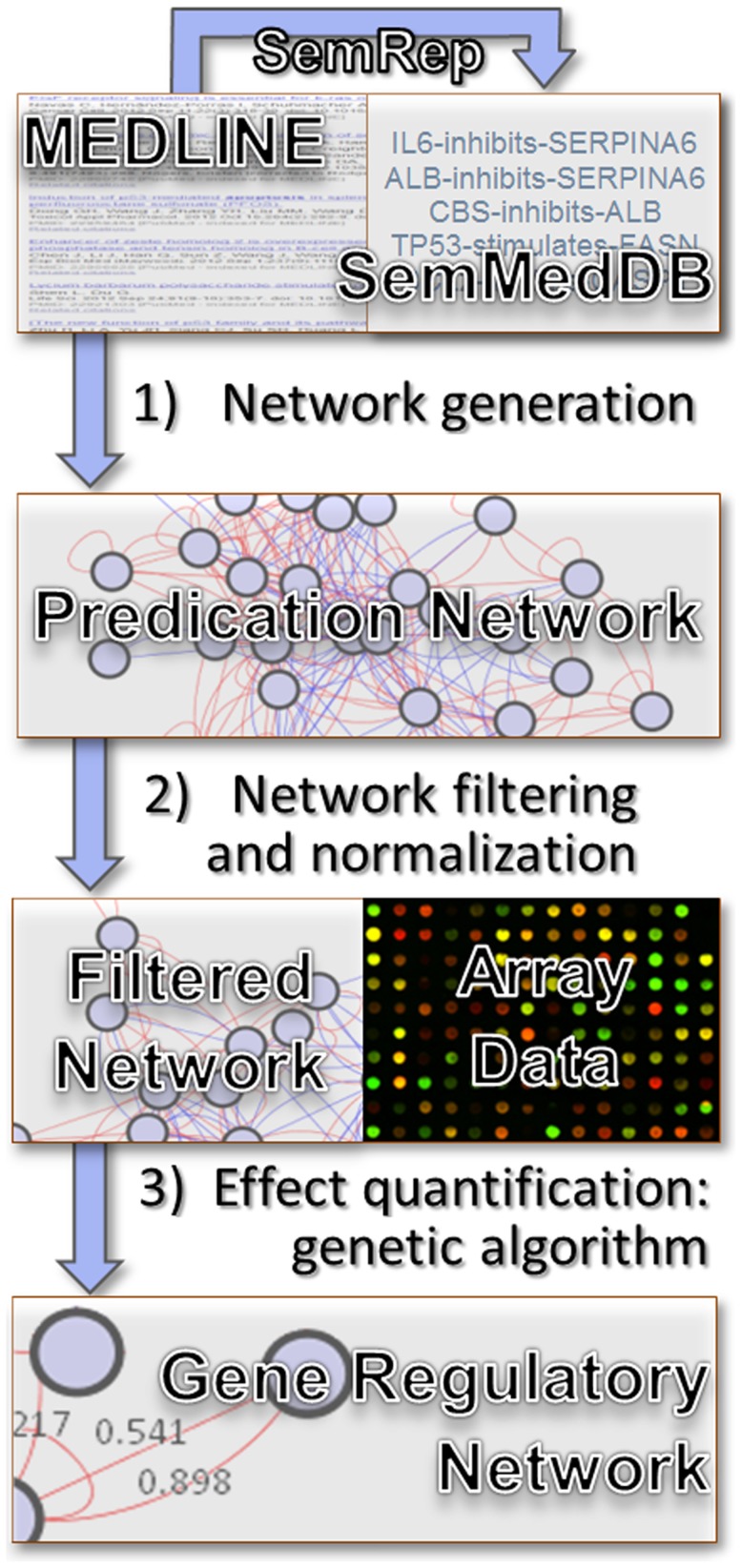
Literature-based gene regulatory network discovery schema. Step 1) a network is formed from semantic predications extracted from MEDLINE by SemRep for each set of citations related to a given pathway; Step 2) each network is filtered by predication argument distance and frequency; Step 3) a genetic algorithm uses gene expression data to quantify the weight of the interactions of the gene regulatory network.

We infer gene regulatory networks in three steps, illustrated in Figure 1.

- *Network generation* relies on SemRep and results in an initial predication-based network.
- *Network filtering and normalization* involves normalizing gene names to formal gene symbols and selecting the most reliable interactions from the predication network.
- *Effect quantification* uses a genetic algorithm with an ANN fitness function on microarray data to quantify gene-gene interaction strength in the network created in previous steps.

**Figure 2:**
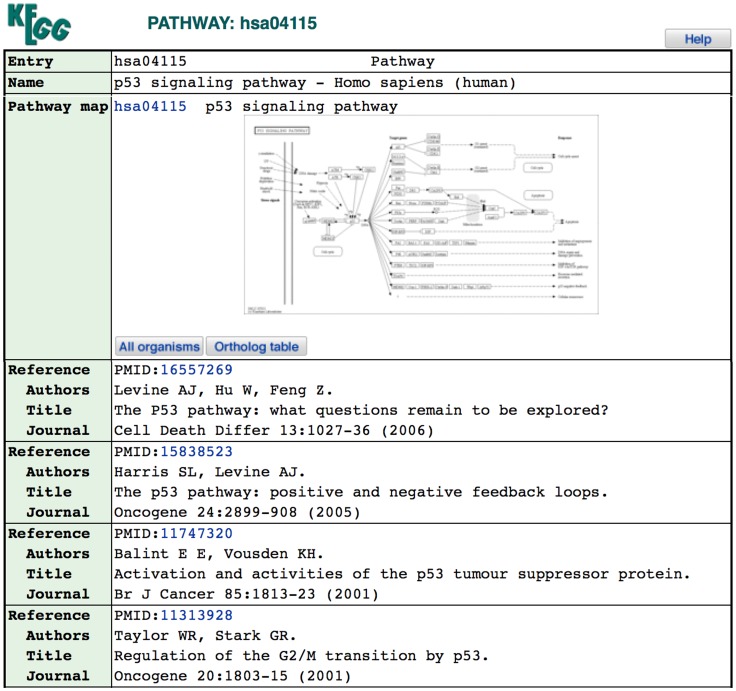
The KEGG *p53* signaling pathway entry showing the pathway map and some reference citations. The PMIDs for each reference citation were used to identify MeSH terms with PubMed (see section *Predication network generation*). http://www.genome.jp/kegg-bin/show_pathway?hsa04115.

To facilitate the establishment and assessment of the system model, we select the well-studied disease breast cancer as the starting point, due to the availability of relevant citations, microarray data, and established KEGG pathways. The 13 pathways contained in the KEGG Pathways of Cancer (human) map (http://www.genome.jp/kegg-bin/show_pathway?hsa05200) were used to guide the predication network generation and also in evaluating the resulting GRNs. These pathways are the *p53* (see Figure 2), *Apoptosis*, *Cell Cycle*, *PPAR*, *VEGF*, *MAPK*, *Wnt*, *TGF-beta*, *mTOR*, *jak-STAT*, *ErbB*, *Focal Adhesion*, and *Adherens Junction* pathways.

### Predication network generation

In the first step, predication network generation, predications containing gene-gene interactions (INTERACTS_WITH, STIMULATES, and INHIBITS predications) are extracted from the MEDLINE citations that supported manual curation of each KEGG pathway; these citations are listed in the pathway entry on the KEGG website. These predications are augmented with those from additional relevant citations, which we identify by first extracting the Medical Subject Heading (MeSH) terms for each citation identified in KEGG and then manually refining that list to eliminate non-specific terms such as Humans, Male, or Biological Models. The resulting MeSH terms formed the basis of our PubMed searches. The terms that occurred in a significant distribution across the citations were grouped with an “OR” in the query when they were roughly equivalent or part of a set of different subtopics. As an example, the query for *p53* citations was “*(Tumor Suppressor Protein p53[mh] OR Genes, p53[mh]) AND (Apoptosis[mh] OR Signal Transduction[mh] OR (Phosphorylation[mh] AND (Neoplasms[mh] OR Neoplasm Proteins[mh] OR Tumor Markers[mh]))) AND physiology[sh] AND metabolism[sh]*.” This procedure was repeated to provide a citation list for each pathway. The number of citations for each pathway is given in Table 1 in ‘Citation’ column. The predications extracted from these citations were then retrieved from SemMedDB. The number of resulting predications for each pathway is shown under the column heading ‘Raw’ in Table 1.

**Table 1:** Number of predications for each pathway.

Pathway	Citations	Predications				
		Raw	Dist.	Uniq.	Norm.	Freq.
p53	5726	14085	3747	2771	1287	155
Apoptosis	13797	27421	6183	5028	1382	93
Cell Cycle	40328	96696	26944	21367	7825	822
PPAR	7701	6730	1708	1388	298	14
VEGF	6102	7875	2416	1940	609	62
MAPK	41455	84611	23341	18639	5859	538
Wnt	2236	2013	685	581	181	8
TGF-beta	14868	23803	7136	5427	1620	126
mTOR	2447	2952	857	740	175	4
Jak-STAT	1121	3193	868	741	275	14
ErbB	4400	7947	2162	1864	710	67
Adherens Junction	6397	6616	1673	1334	275	3
Focal Adhesion	1169	2227	563	510	91	0

Citations: the number of citations returned from the PubMed search using related MeSH terms. Raw: the number of gene interaction predications SemRep extracted from the returned citations. Dist.: the predications after filtering out predications with distance >1. Uniq.: the number of unique predications after eliminating duplicates. Norm.: the number of predications after normalizing against the HUGO database. Freq.: the number of predications after filtering for a frequency of at least 2 predications.

### Network filtering and normalization

**Figure 3:**
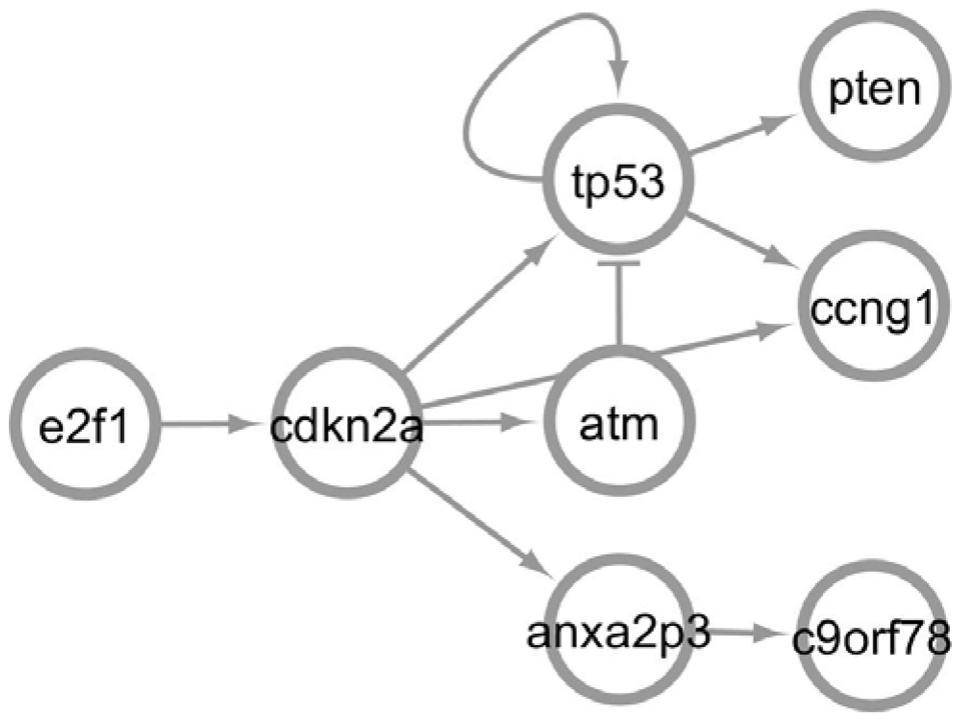
Example network created from semantic predications, edited for simplicity. Arrows indicate stimulation; bars indicate inhibition; loops indicate self-stimulation or self-inhibition. Interactions are directed, so an interaction from atm to tp53 is distinct from one from tp53 to atm. A single interaction can represent a single or multiple predications.

To improve the reliability of our approach, we used three predication filtering mechanisms, explained below. These mechanisms result in a smaller set of predications extracted for each pathway, which forms subnetworks. These subnetworks, together, serve as the predication network. Figure 3 provides an example of a subnetwork created from predications related to *p53*. This example has been pruned to provide a small network for simplicity.

### Argument-predicate distance filtering

Argument-predicate distance is the number of intervening noun phrases between an argument and its predicate in the sentence from which they were extracted. It has been shown that smaller argument distance leads to higher precision in extracting interactions [62]. In the example below, both *Bax* and *pro-caspase-3* are potential objects for *p53* activity.

3. Furthermore, the up-regulation of *p53* promoted *Bax* expression, which led to the activation of *pro-caspase-3* and eventually to apoptosis in MCF10A-ras cells. (PMID: 5566875)

*Bax* has an object distance of 1 since it is the first noun phrase subsequent to the predicate ‘promoted’ and *pro-caspase-3* has a distance of 2. Preferring an argument-predicate distance of 1 selects the predication *p53* STIMULATES *Bax*, while eliminating the predication *p53* STIMULATES *pro-caspase-3*. In our experiments, we limit both the predicate-subject distance and the predicate-object distance to 1, which decreased the number of predications to approximately 27% of the initial number of predications on average. The number of predications for each pathway after filtering using argument-predicate distance of 1 is shown in Table 1 (under ‘Dist.’).

### Normalization

After argument-distance filtering is applied, we normalize the set of predications by removing duplicate predications and mapping the subjects and objects to formal gene symbols based on the standard gene name dataset (HUGO, http://www.genenames.org/). If a predication argument cannot be mapped to a formal gene symbol, the predication is pruned. The numbers of predications after duplicate removal and normalization are given in Table 1 (under ‘Uniq.’ and ‘Norm.’, respectively).

### Document frequency filtering

Document frequency for a predication is the number of citations in which the predication occurs. Document frequency filtering is based on the hypothesis that the confidence credential of a predication is in direct ratio to its occurrence in documents. In our experiments, we discard all predications that occur in fewer than two articles. The result is shown in Table 1 (‘Freq.’). Note that network filtering discards all relevant predications for the *Focal Adhesion* pathway, which was not considered in subsequent steps.

### Effect quantification

**Figure 4:**
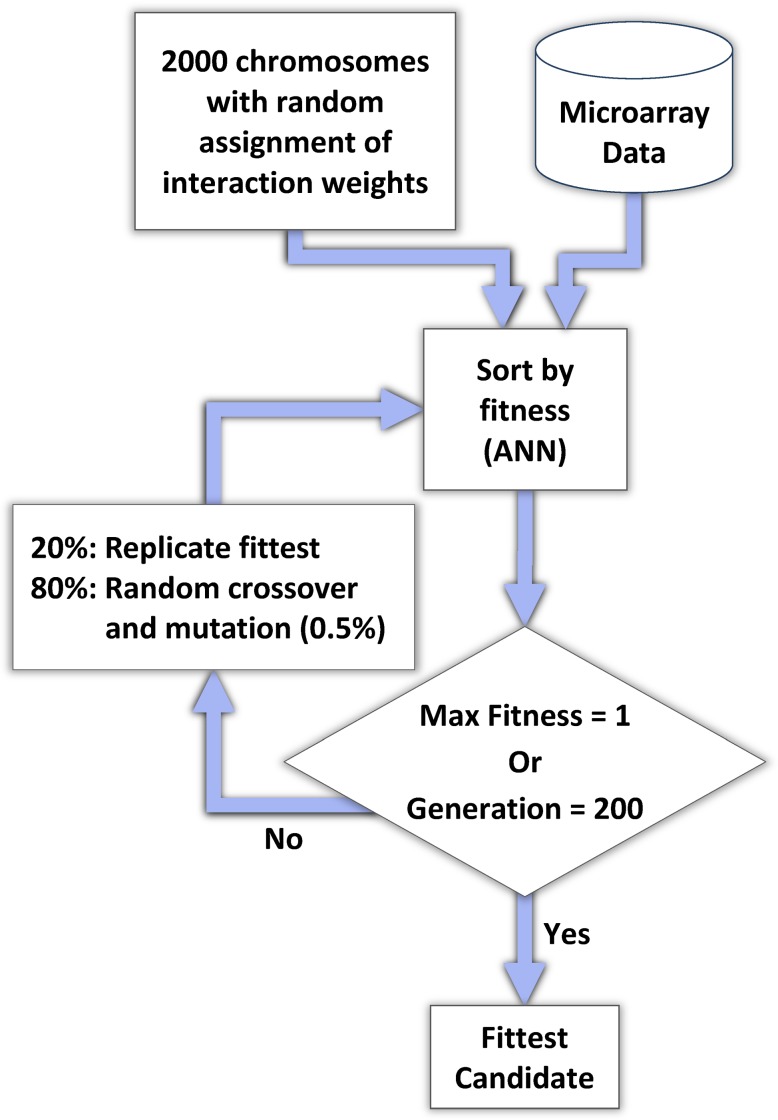
Genetic algorithm for effect quantification. An initial set of 2000 chromosomes, each containing the full complement of genes paired with every other gene, is created with an initial random assignment of interaction weights. The fitness of a chromosome is compared against the expression profile in the microarray data. The fittest 20% of chromosomes are replicated into the next generation, and the complement of the population is formed by random crossover and point mutation of interaction weights of members of the current generation. After 200 generations, the fittest candidate chromosomes are identified. ANN = artificial neural network.

In the final step we quantify gene-gene interaction strength in the network generated in previous steps, using a genetic algorithm, depicted in Figure 4. In the initial population of chromosomes, each chromosome contains a candidate set of interactions between all genes in the pathway. The predication network determines the initial gene-gene interactions, while the strength of interaction is initially randomly generated for each of the 2000 chromosomes in the population. Then for each generation, the fittest candidates are replicated into the next generation and the balance of the population is filled through reproduction of the current generation, using random crossover and mutation to introduce novel diversity into the population. We compute the fitness of a chromosome, as compared to the time series microarray data, using an artificial neural network. These procedures are described in more detail in the following subsections.

### Genetic algorithm

With the genetic algorithm used to infer the gene regulatory networks, we train the weights of the inbound interactions for every gene separately. A population of chromosomes is created where each chromosome is represented as a matrix of interaction weights between each possible pairing of genes identified from the predications. Each weight, valued between −1 (greatest inhibition) and +1 (greatest stimulation), indicates the strength of interaction. A weight of zero indicates no interaction. The predications in the network define which genes have interactions and whether the interaction is inhibitory or stimulatory, but do not contribute to the determination of the weight. The absence of an interaction is represented in the matrix with a weight of zero but is not altered through subsequent generations. The direction of the interaction is maintained from the subject-object relation in the predication. We randomly generate 2000 chromosomes that contain different weights for the interactions in the predication network. The gene structure in each chromosome is the same, as well as the non-interacting/zero-weighted gene pairs, but the weights representing the strength of inhibition or stimulation of pairs found in the predications are varied. The population of chromosomes is then evaluated with the fitness function in Equation 1, and the fittest 20 percent of the chromosomes are copied into the next generation directly. The rest (80 percent) of the chromosomes for the next generation are generated by crossover at a specific (randomly selected) gene pairing between pairs of randomly selected chromosomes in the current generation with a mutation rate of 0.25 percent. This mutation rate approximates those commonly used to facilitate convergence of the chromosomes toward a fittest result and to avoid excessive intergenerational fluctuation. After evolving for 200 generations (an empirically determined limit), the chromosome with the highest fitness value is selected as the final result.

### Fitness test function

**Figure 5:**
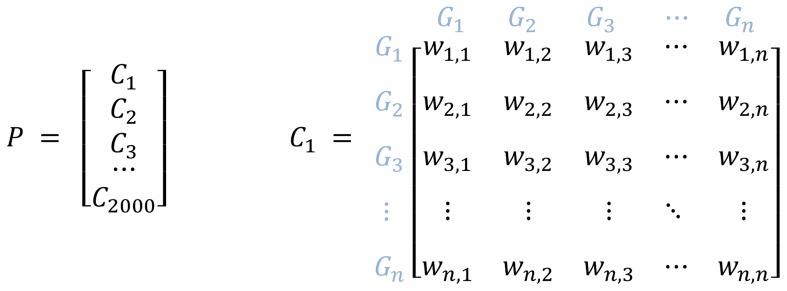
Population and chromosome weight matrix. A population of 2000 chromosomes is initially generated and maintained at each new generation. Each chromosome contains a matrix of interaction weights between every pairing of genes. *P* = population, *C* = chromosome, *G* = gene, *w_2,1_* = the weight of the interaction from gene 1 to gene 2.

**Figure 6:**
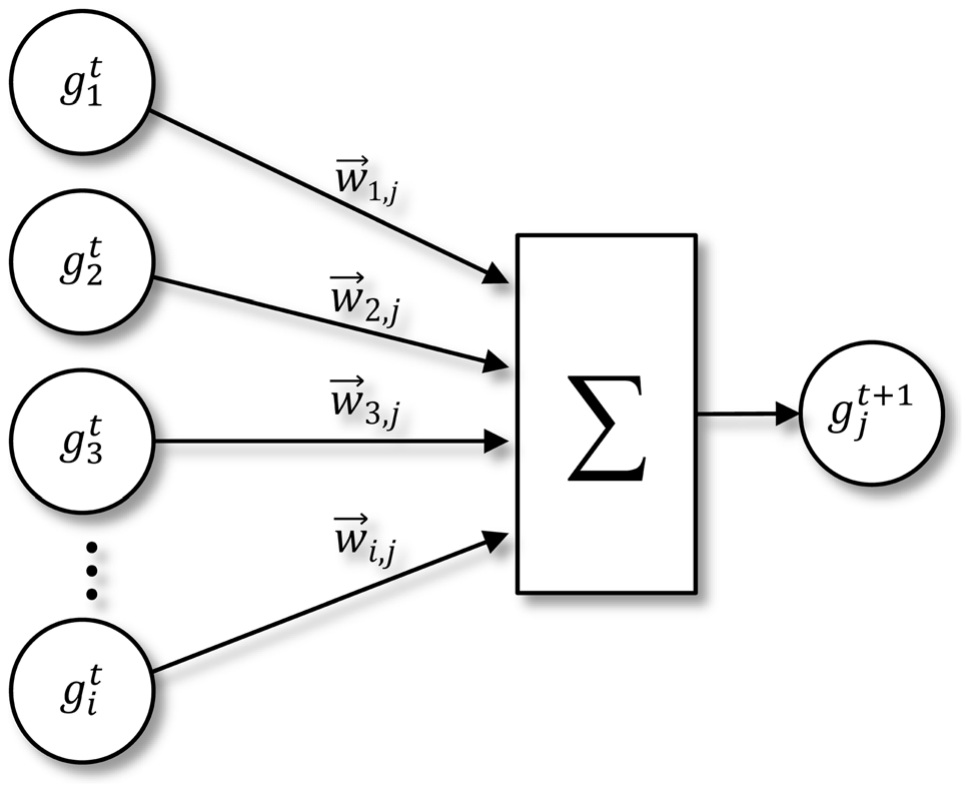
Artificial neural network fitness function. Each contributory gene's expression in the current time point is magnified by its weight of interaction with the target gene. The sum of these contributions determines the expression level of the target gene at the subsequent time point. *t* indicates the time point in the microarray dataset, *n* is the number of genes in the pathway, 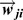 is the weight of the interaction between gene *i* and gene *j*, 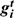 is the gene expression value in the microarray set for gene *i* at time point *t*.

In our experiments, we use an artificial neural network (ANN) to model the interaction of the genes in each pathway. In this model, the gene expression level of a given gene at a given time point is a function of all other genes at the previous time point (see Figure 5). In a comparative study by Sîrbu et al., this model outperformed 4 other recent GRN models (GA + evolutionary strategy, differential evolution + Akaike's Theoretic criterion, genetic local search, and an iterative algorithm based on GA) in a combined score of 6 performance measures (data fit, parameter quality, noise, sensitivity, specificity, and scalability) [10]. We follow the most common implementation of such a model, using a nonlinear weighted sum. Equation (1) shows the fitness test function based on the ANN model.
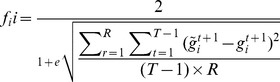
(1)where
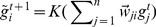
(2)and *T* indicates the number of time points in the microarray dataset, *R* is the number of microarray replicates at each time point, *r* is the current replicate, *n* is the number of genes in the pathway, *K* is the activation function, 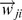 is the weight of the interaction between gene *j* and gene *i*, and 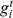 is the gene expression value in the microarray set for gene *i* at time point *t*. Since the difference of the expression value of a gene over each time course is considered to be a direct function of only the joint effect of other genes in the pathway and the gene expression values used have been normalized between −1 and 1, we use a linear function as the activation function, effectively dropping the term. A visual representation of the ANN model is presented in Figure 6.

### Inference of yeast cell cycle GRN

MEDLINE contains many more citations related to human genes than yeast genes. A simple PubMed search for “yeast and gene” returns a little more than 86,000 citations while the query “human and gene” returns over a million. When combining either species term with “cell cycle” (a concept with a heavy amount of research done in yeast), there are over 96,000 citations related to human and just over 15,000 for yeast. In addition to this limitation, SemRep was designed to identify human genes and uses Entrez Gene database entries specific to humans for genes and proteins. Although this led to an initial study with human genes, we made modifications to SemRep to support a study with yeast. This required changing the data source to the Entrez Gene fungi dataset that contains *Saccharomyces* species and processing relevant citations (resulting from the PubMed query “Saccharomyces[mh] AND cell cycle[major]”) to extract predications. All subsequent procedures were consistent with the breast cancer study except that the predications were not filtered by argument distance or document frequency due to the lower initial number of predications. The number of citations and predications at each step are given in Table 2.

**Table 2:** Number of predications for yeast cell cycle pathway.

Citations	Predications		
	Raw	Unique	Normalized
3480	1567	1387	260

Citations: the number of citations returned from the PubMed search using related MeSH terms. Raw: the number of gene interaction predications SemRep extracted from the returned citations. Unique: the number of unique predications eliminating duplicates. Normalized: the number of predications after normalizing against the Entrez database.

## Results

Evaluating a gene regulatory network inference model for human data is challenging, since there is no gold standard providing a complete reference of gene connectivity. Therefore, we evaluated the implementation of our approach using breast cancer in two ways: (1) comparing the accuracy of the model against the highest-performing model reported in the literature, (2) comparing the resulting networks to KEGG pathways and literature that formed the basis for their extraction, paying particular attention to the *p53* pathway. Comparison to a KEGG pathway provides an assessment of the contribution of our natural language processing techniques (i.e., SemRep).

### Comparison of model accuracy

**Figure 7:**
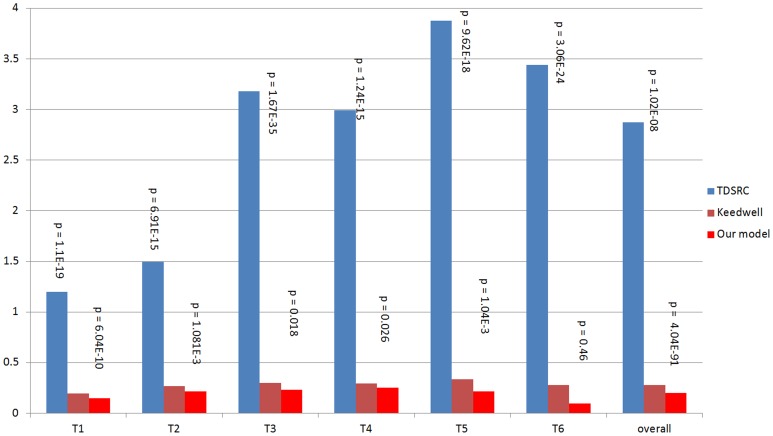
Comparison of fitness to microarray data. Fitness of our model is compared to that of the Keedwell et al. model and the TDSRC model on our microarray dataset. Fitness is significantly better (p<0.05) in 4 out of 6 time points and for all time points combined (overall).

As a measure of accuracy of the model, we determine how well the model fits the data, in line with previous research [21]. For this purpose, we use microarray data from a human breast cancer experimental set (GEO: GSE29917), which contains expression values for 7 time points and 6 replicates (two microarrays are missing from the set for a total of 40 microarrays). We compare the accuracy of our model with that of the Repeated Genetic Algorithm with Neural Network model described by Keedwell et al. [21], the best performing model in a recent comparison of evolutionary algorithms [10] and the basis for the interaction quantification component of our algorithm. Since we use the same algorithm for interaction quantification, the comparison helps isolate the effect of literature-derived knowledge on GRN inference. We downloaded their source code to be able to run their algorithm on our data, with only minimal modification for data format differences. Additionally we included a second comparison model, with a different type of algorithm (time-delayed Spearman Rank-correlation or TDSRC) to help identify any biases in our methodology [63]. This model was included by Gupta et al. as a part of a composite model and available for download and use within MatLab. For this comparison, we limit the microarray data to 78 genes included in the KEGG P53 pathway (listed in Table 3). We use a leave-one-out approach based on time points, sequentially using gene expression values for each time point as test data against models trained with values from all of the other time points. Fitness is defined as the standard deviation of predicted values from the average microarray expression of all 6 replicates in the test set. Because the microarrays for 2 different replicates at two different timepoints were missing, the total number of microarrays was 40. The root mean square error (RMSE) combines fitness for all genes in the given test set. Figure 7 shows the root mean square errors over the 78 genes for each time point for each model. We used a paired t-test to assess the statistical significance of the differences between models. The p-values for each comparison are included in Figure 7. Our model shows improvement at every test data set/time point over the Keedwell et al. model and is significantly different (p<0.05) in 4 out of 6 time points and for all time points combined (overall p = 8.26×10^−8^). The RSP model performed significantly worse than both of the other models. This demonstrates that our model is significantly better in terms of fitting the microarray data.

**Table 3:** Genes from KEGG P53 Pathway.

cdkn2a	casp1	cntn2	foxn3	mdm4	psmd10	tnfsf10
apaf1	casp8	crk	gadd45a	mtor	pten	tp53
atm	ccnb1	csh2	h2afx	myc	rchy1	tp73
axin1	ccnd1	daxx	hipk2	mycn	rhd	ube2s
bak1	ccng1	e2f1	igfbp3	nanog	s100b	ybx1
bax	cdk2	efemp1	ing1	parp1	serpina2	znf331
bcl2	cdk5	egf	jun	pcna	sirt1	
bcl6	cdk9	eif2ak2	mapk1	pin1	stat3	
bik	cdkn1a	ep300	mapk14	pmaip1	stk11	
birc5	cflar	epha2	mapk8	pml	tnf	
brca1	chek1	ets1	mapk9	pold1	tnfrsf10a	
bub1	chek2	fasn	mdm2	ppm1d	tnfrsf10b	

List of genes included in the KEGG P53 pathway.

### Assessment of interactions

We assessed the value of gene regulatory networks generated by our approach by comparing them to KEGG pathways, both the kgml format downloaded from the KEGG website and manually using the search function in the online pathway map. Nodes, representing genes, and edges, representing interactions, were independently compared and the results are shown in Table 4. The ‘Predication’ column represents the number of nodes and edges in the network generated by our approach after removing any genes that were not included in the microarray set, the ‘KEGG’ column provides the equivalent information for the kgml network, ‘Common’ indicates the intersection of our results and the corresponding kgml network, and ‘New’ provides interactions exclusive to our results. As the numbers indicate, new nodes and edges significantly outnumber those in common between the two networks. The *p53* pathway had the highest ratio of common edges compared to the kgml network (19∶65, 29.2%), so was chosen for further validation at the predication level.

**Table 4:** Comparison of resulting network and kgml network.

Pathway	Predication		KEGG		Common		New	
	Nodes	Edges	Nodes	Edges	Nodes	Edges	Nodes	Edges
P53	78	110	55	65	23	18	55	92
Apoptosis	57	69	45	62	12	2	45	67
Cell Cycle	341	594	76	182	44	24	297	570
PPAR	7	10	47	112	3	0	4	10
VEGF	44	57	28	34	7	1	37	56
MAPK	210	432	118	168	49	18	161	414
Wnt	9	7	57	70	4	1	5	6
TGF-beta	72	97	43	63	12	3	60	94
mTOR	8	6	21	25	3	2	5	4
Jak-STAT	14	14	22	27	1	0	13	14
ErbB	31	44	47	118	14	25	17	19
Adherens Junction	6	3	48	83	1	0	5	3

Predication: number of nodes and edges in the predication network; KEGG: number of nodes and edges in the KEGG network; Common: number of nodes and edges in both predication and KEGG pathway; New: number of nodes and edges found in predication but not KEGG network.

A comparison was made for each of the new interactions with the online KEGG *p53* signaling pathway map. 49 of the 92 interactions contained a gene not included in the map, 7 had both genes present but no interaction, and 9 existed in the map but were not included in the kgml version.

**Figure 8:**
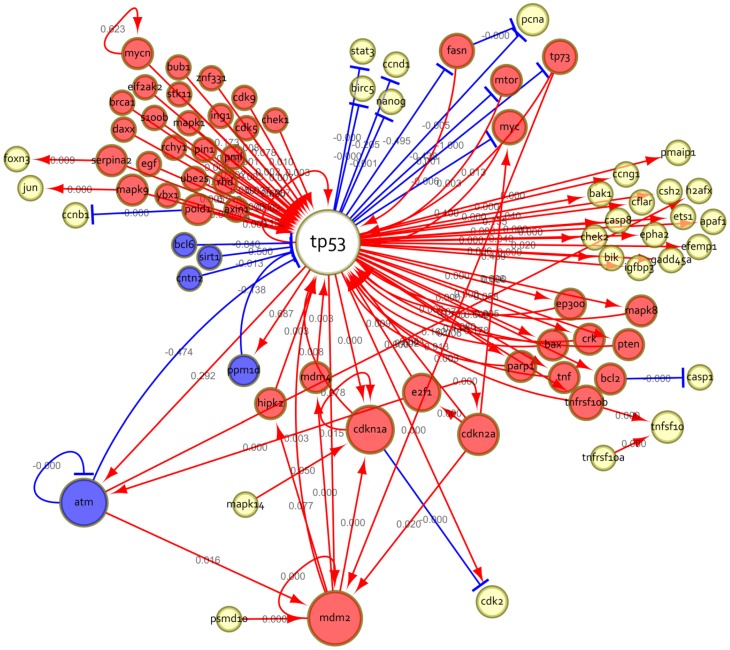
The resulting gene regulatory network for the *p53* pathway. Interactions are defined by predications extracted from MEDLINE citations and weighted based on microarray data. Red node color signifies induction or activation of *p53* while blue signifies suppression or deactivation and yellow indicates genes that do not act directly onto *p53*. Red arrows show induction/activation. Blue arrows show inhibition. Size of a node corresponds to the number of connections to other nodes.

We also validated each new interaction in the resulting *p53* network against the literature in three ways: a) whether the literature asserts an interaction between the two at either a gene or protein level (i.e., the precision of our natural language processing techniques), b) whether we capture correctly the direction of the effect, i.e. stimulatory or inhibitory, as compared to the weight generated by GRN analysis of the microarray data, and c) combining the two above, whether we capture correctly both the presence of the interaction and the direction. In addition to assessing the accuracy of SemRep in capturing relevant interactions, this validation allows us to establish the specific contribution of using semantic predications over term co-occurrence. The comparison was limited to citations from which the predications were extracted, ranging from 1 to 87 citations for each interaction. Although there may exist another citation that would validate the resulting interaction, this approach was taken to limit the man-hours required to a reasonable amount while still allowing a reasonable possibility for validation. As seen in Table 5, 78.3% of the new interactions in the resulting *p53* network (Figure 8) were asserted in at least one of the source citations. When comparing the sign of the interaction weight to the direction of the effect provided in the source literature, 76.4% were consistent. Those interactions that were both consistent with the literature and were weighted in the appropriate direction numbered 55 out of 92 (59.8%).

**Table 5:** Literature validation of newly included interactions.

	Validation Type	Count
All results	Interaction stated	72 (78.3%)
	No interaction stated	20 (21.7%)
	Correct sign	75 (76.4%)
	Inverse sign	17 (23.6%)
	Correct sign and interaction	55 (60%)
	Wrong sign or interaction	37 (40%)
Weights >0.1	Interaction stated	32 (84.2%)
	No interaction stated	6 (15.8%)
	Correct sign	34 (87.5%)
	Inverse sign	4 (12.5%)
	Correct sign and interaction	28 (73.7%)
	Wrong sign or interaction	10 (26.3%)

Each interaction was evaluated against source literature to evaluate its accuracy. Evaluation included whether an interaction between the indicated genes was actually contained in the sentence, and whether the resulting sign (−/inhibits or +/stimulates) corresponded to the assertion in the sentence. The upper half includes every interaction regardless of weight, whereas the lower half refers to only interactions with weight >0.1.

We additionally focused on those interactions having a weight with absolute value >0.1 (Table 5, bottom), exploring the hypothesis that stronger interactions should be less affected by noise. Within these interactions a total of 32 interactions (84.2%) were stated in the literature and 34 (87.5%) were correct in the direction of effect, yielding 28 (73.7%) correct on both counts.

Finally, we investigated whether argument-predicate distance filtering had a detrimental effect on the results, since long distance syntactic dependencies are common in biomedical literature [64]. For this purpose, we focused on the Jak-STAT pathway and checked whether any of the 27 KEGG interactions, none of which appeared in our predication network, could be derived from the initial set of unfiltered, non-normalized predications. We found three such predications for the Jak-STAT pathway; one (*Jak1 INTERACTS_WITH PTPN11*) was eliminated due to argument-distance filtering, while the other two (*Jak1 INTERACTS_WITH STAT1* and *GRB2 INTERACTS_WITH PTPN11*) passed the argument-distance filter but not the document frequency filter. Note that the former (*Jak1 INTERACTS_WITH PTPN11*) occurred in a single document and, therefore, would also be eliminated in the subsequent document frequency filtering step, had it passed the predicate-argument distance filter.

### Performance on yeast data

**Figure 9:**
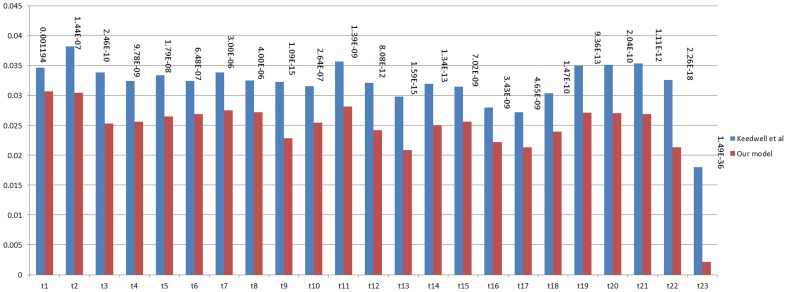
Comparison of fitness to yeast cell cycle microarray data. Fitness of our model is compared to that of the Keedwell et al. model on a yeast cell cycle microarray dataset. Fitness is significantly better (p<0.05) over all points.

We compared the performance of our model against that of Keedwell et al. in the same manner as mentioned in our results section *Comparison of model accuracy*, but now on the yeast cell-cycle dataset, which contains 24 time points. Figure 9 shows the root mean-square errors for each time interval for the two models. We assessed statistical significance of the differences between models by calculating the p-values (included in Figure 9) using a paired t-test. Using the yeast data, our model had increased fitness at every interval over the Keedwell et al. model, but this time every difference was significantly different (p<0.05).

## Discussion

In this work, we augmented microarray data with literature knowledge to infer gene regulatory networks, replacing the random selection of component genes generally used in similar modeling efforts. We use SemRep to extract information from the literature, which forms the backbone of the network. State-of-the-art genetic algorithm-based analysis of the microarray data was used to determine the strengths of the effects between gene-gene interactions in the network. Our model provides better fitness than the state-of-the-art model used as the basis for the genetic algorithm and fitness function components of our model and the difference between error rate of the models is statistically significant (p<0.05), demonstrating that literature-derived knowledge provides a significant advantage over random selection of genes in this task, as suggested in [10]. Another advantage of our approach is that it maximizes the best possible networks without being limited to relationships that are already well established and considered important by a curator, as would be the case if a standard interaction database were used. These pathways provide targets for novel therapeutic interventions and a mechanistic understanding of current therapeutic approaches with poorly understood mechanisms.

### Interactions identified by our model not included in KEGG

Although some of the genes in the KEGG pathways did not appear in the results due to the strict filtering process, the presence of essential member genes in the resulting predication networks (for example, *TP53*, *MDMD2*, *BAX*, *CDKN1A*, *GADD45A*, and *CDK2* in the *p53* network) and known interactions (*p53 STIMULATES Gadd45a*, *E2F1 STIMULATES cdkn2a*, and *RCHY1 INHIBITS tp53*) demonstrate the potential of this technique to replicate “known” pathways. Perhaps more importantly, we were able to identify new interactions that were not included in the KEGG maps, which is not surprising since these maps are curated and therefore provide only the most thoroughly established and important interactions as determined by the curators.

Two of the highest-weighted new interactions included in the resulting *p53* pathway but not in the kgml file are *p53 STIMULATES BIK* and *MDM2 INHIBITS CDKN1A*. The weight of the interaction between *p53* and *BIK* was very strongly stimulatory at 0.977. *BIK* (*BCL-2* interacting killer) is not included in the KEGG *P53* pathway map. It is a pro-apoptotic protein discovered in 1995 and interacts strongly with *BCL-2* and *BCL-xL* [65]. *P53* has been shown to induce expression of *BIK* under certain conditions, especially in breast cancer cells, as in our microarray dataset [66]. Our *p53* pathway result also included an interaction between *MDM2* an *CDKN1A* with a weight of −0.987, i.e., very strongly inhibitory. *MDM2* is shown to inhibit *p53* in the KEGG *p53* map but there is only an indirect inhibitory action of *MDM2* on *CDKN1A* through *P53* (by diminishing *p53*'s activation of *CDKN1A*). However, a direct interaction as a negative regulator of *CDKN1A* is present in the literature [67], supporting our result.

### Limitations and future directions

A major limitation of this method is the accumulation of system errors. As shown in Table 5, the accuracy of interactions identified using SemRep with the filtering used in our method is 78.3% overall and 84.2% for significantly weighted interactions (>0.1). The reproducibility of the microarray data between platforms and even with different algorithms on the same platform has been determined to be as low as 50–60% [68]. At a result, the accuracy of both the structure and the weights of the resulting GRNs is limited if viewed at the most precise level of granularity. The training of the weights is also limited by an insufficient time course in the microarray data, especially for large numbers of arguments (i.e. genes in a pathway). Although it is beyond our means to improve microarray reproducibility or experimental design of publicly available datasets, improvements to SemRep with regard to gene and protein interactions can potentially improve results and the incorporation of multiple microarray datasets as training data may be able to overcome some of the inherent limitations. Additionally, although these limitations affect the use of these techniques for determining the precise quantitative nature of interactions, this is true generally of such studies, and does not prevent their use for hypothesis generation.

Currently our network backbone is limited to genes included in the predications, but this set could be expanded by incorporating genes from the microarrays that are similar to the genes provided by the predications, using an ensemble of machine learning techniques such as support vector machines, random forests, and prediction analysis of microarrays. This approach of classifying genes from the microarray into each pathway would additionally reduce bias in the technique by not limiting interactions to what is already known. It would be valuable to maintain a distinction between the literature-based genes and the expansion set because a significant expansion in the final resulting networks would suggest that what is known in these pathways is only a fraction of what is waiting to be discovered.

Another technique to extend the set of genes would be to adopt more sophisticated predication filtering mechanisms. The current mechanisms were effective in significantly reducing the computational complexity; however, our limited analysis of the effect of argument-distance filtering showed that some relevant predications were also eliminated due to filtering. A potentially useful approach would be to train a classifier that can identify ‘good’ predications, based on predication features, such as the predicate type, indicator types, etc. Predicate-argument distance could also serve as a feature for such a classifier.

Additionally, although we used interactions from KEGG to evaluate our GRN, such interactions from this and similar databases could be incorporated into our interaction network to augment the proposed interactions from literature with established interactions, expanding the utility of prior knowledge.

Although our current method of selecting source citations for predication extraction yielded usable results, there are many possible permutations in the method and a systematic comparison of source citations and their resulting networks should be explored. Our current search method facilitates the generation of hypothetical member genes for established pathways, but this approach can also be used to generate novel pathways by specifying a gene set and/or biological functions in the predication citation search, thereby providing an appropriate predication network.

SemRep representation of biomolecular interactions (subject-predicate-object triples) is simple, intuitively accessible and lends itself easily to downstream applications. On the other hand, more complex representations, particularly event representation (as discussed in *Related work* section), have been gaining in popularity in the BioNLP community, mostly due to available corpora [69] and shared task competitions [27]. An obvious question is whether such complex representations could provide an advantage over or could complement SemRep representation in the task of gene regulatory network inference. It seems very likely that generating a seed network from more complex representations would require some non-trivial post-processing along the lines of the algorithm described by Bossy et al. [36]. Since that algorithm essentially breaks down complex relations to simpler SemRep-style triples, it seems safe to assume that SemRep representation can adequately capture the complexity of biomolecular interactions. However, this needs further testing and validation.

To assess the precision of interactions for the yeast study, we performed an automated comparison using Cytoscape (http://www.cytoscape.org) against a Biogrid (http://thebiogrid.org) yeast interaction dataset. We used the Biogrid Saccharomyces_cerevisiae-3.2.109 tab file, which contains 339,921 interactions and experimental information from multiple database sources including Saccharomyces Genome Database, MINT, IntAct, and Pathway Commons. As seen in Table 6, although 346 out of 349 genes are found in the reference set, only 147 out of 520 interactions are included. That is a relatively low precision of 28%, which only increases to 31% for interactions weighted 0.1 or higher. This is not particularly meaningful without appropriate context. Since this is a relatively straightforward and easily undertaken evaluation approach, it would be worthwhile to conduct a study applying the same evaluation across various published models to see how precision compares among them.

**Table 6:** Precision of yeast interactions.

Weight threshold	Predicted		In Biogrid		Precision	
	Genes	Interactions	Genes	Interactions	Genes	Interactions
0	349	520	346	147	0.991	0.283
0.001	349	300	346	90	0.991	0.300
0.01	349	244	346	72	0.991	0.295
0.1	349	67	346	21	0.991	0.313

Each gene and interaction predicted by our model was compared against interactions contained in the Biogrid dataset. Predicted: number of genes and interactions predicted by our model. In Biogrid: number of genes and interactions predicted by our model and found in the Biogrid dataset. Precision: number found/number predicted.

### Conclusions

We present a methodology of gene regulatory network inference that combines literature knowledge and microarray data. Using SemRep, we extract gene and protein interactions from citations related to the pathways included in the KEGG Pathways of Cancer. These predications are linked together to form a network and a genetic algorithm is used on a breast cancer time sequence microarray dataset to determine the weight of contribution of each stimulating or inhibiting gene on its target, thereby providing a weighted gene regulatory network. Our model performs significantly better than comparable models in terms of fitness of predictive output to microarray results. The resulting networks contain both interactions included in the appropriate KEGG pathways and interactions not included but validated through a literature search. The accuracy of these interactions increases from 60% overall to 74% when minimally-weighted interactions are excluded. Our model also performed better in terms of fitness against a comparison model when modified for yeast predications and microarray data. This approach offers significant potential in elucidating new interactions in existing pathways as well as the possibility of identifying novel pathways. In a broader sense, it also provides a blueprint for incorporating automatically extracted knowledge from literature with large-scale biological analysis. Incorporating such knowledge underpins more accurate understanding of both normal and disturbed molecular physiology, leading to advances in diagnosis and treatment of disease.

## Supporting Information

Table S1 **PubMed queries for each pathway.** The query submitted to PubMed for each pathway in the breast cancer study is provided. Each search provided a citation list relevant to the 13 pathways in the KEGG Pathways of Cancer map. mh: MeSH heading. sh: subject heading.
(DOCX)
