# Supplementary material for: Augmenting Microarray Data with Literature-Based Knowledge to Enhance Gene Regulatory Network Inference
Source: PLoS Comput Biol. 2014 Jun 12;10(6):e1003666. doi: 10.1371/journal.pcbi.1003666 (PMC4055569; doi:10.1371/journal.pcbi.1003666)
Supplement: Table S1 — PubMed queries for each pathway. The query submitted to PubMed for each pathway in the breast cancer study is provided. Each search provided a citation list relevant to the 13 pathways in the KEGG Pathways of Cancer map. mh: MeSH heading. sh: subject heading. (DOCX) [file pcbi.1003666.s001.docx]

**Supplementary Table 1 – PubMed queries for each pathway**

| **Pathway** | **Query** |
| --- | --- |
|  |  |
| Adherens Junction | (Cell Adhesion Molecules[mh] OR Cadherins[mh]) AND (beta Catenin[mh] OR Cell Movement[mh] OR (Cytoskeletal Proteins[mh] AND Trans-Activators[mh])) AND metabolism[sh] AND physiology[sh] AND NOT Integrins[mh] |
| Apoptosis | *Apoptosis[mh] AND metabolism[sh] AND *physiology[sh] AND (Proto-Oncogene Proteins c-bcl-2[mh] OR Caspases[mh] OR Cell Cycle[mh] or Signal Transduction[mh]) |
| Cell Cycle | (Cell Cycle[mh] OR Cell Cycle Proteins[mh]) AND physiology[sh] AND metabolism[sh] AND (Intracellular Signaling Peptides and Proteins[mh] OR DNA-Binding Proteins[mh] OR Tumor Suppressor Proteins[mh] OR Nuclear Proteins[mh] OR Protein Kinases[mh]) |
| ErbB | (Receptor, Epidermal Growth Factor[mh] OR Receptor, erbB-2[mh] OR Receptor, erbB-3[mh]) AND Signal Transduction[mh] AND physiology[sh] AND metabolism[sh] AND (Proto-Oncogene Proteins[mh] OR Neoplasms[mh] OR Endocytosis[mh] OR Phosphorylation[mh] OR Dimerization[mh] OR hosphatidylinositol 3-Kinases[mh]) |
| Focal Adhesion | (Cell Cycle[mh] OR Cell Survival[mh] OR Cell Movement[mh] OR Neoplasms[mh] OR rho GTP-Binding Proteins[mh] OR Phosphatidylinositol 3-Kinases[mh]) |
| Jak-STAT | (Janus Kinase 1[mh] OR Janus Kinase 2[mh] OR Janus Kinase 3[mh]) AND Signal Transduction[mh] AND Cytokines[mh] AND physiology[sh] AND metabolism[sh] |
| MAPK | (map kinase signaling system[MeSH Terms] AND "physiology"[Subheading]) OR (mitogen-activated protein kinases[MeSH Terms] AND ("mitogen-activated protein kinase kinases"[MeSH Terms] OR "map kinase kinase kinases"[MeSH Terms] OR "receptor protein-tyrosine kinases"[MeSH Terms] OR "phosphatidylinositol 3-kinases"[MeSH Terms] OR "dna-binding proteins"[MeSH Terms] OR "signal transduction"[MeSH Terms]) AND physiology[sh]) |
| mTOR | TOR Serine-Threonine Kinases[mh] AND physiology[sh] AND metabolism[sh] AND Signal Transduction[mh] |
| p53 | (Tumor Suppressor Protein p53[mh] OR Genes, p53[mh]) AND (Apoptosis[mh] OR Signal Transduction[mh] OR (Phosphorylation[mh] AND (Neoplasms[mh] OR Neoplasm Proteins[mh] OR Tumor Markers[mh] ))) AND physiology[sh] AND metabolism[sh] |
| PPAR | (Peroxisome Proliferator-Activated Receptors[mh] OR Thiazolidinediones[mh]) AND metabolism[sh] AND physiology[sh] AND ((Gene Expression Regulation[mh] OR Transcription, Genetic[mh]) OR (Glucose Metabolism Disorders[mh] OR Metabolism[mh]) ) |
| TGF-beta | Transforming Growth Factor beta[mh] AND metabolism[sh] AND physiology[sh] AND ((Smad Proteins[mh] AND Trans-Activators[mh]) OR Receptors, Transforming Growth Factor beta[mh] OR Bone Morphogenetic Proteins[mh] OR Signal Transduction[mh] OR Gene Expression Regulation[mh]) |
| VEGF | (Vascular Endothelial Growth Factors[mh] OR Receptors, Vascular Endothelial Growth Factor[mh]) AND physiology[sh] AND metabolism[sh] AND (Neovascularization, Physiologic[mh] OR Signal Transduction[mh] OR Mitogen-Activated Protein Kinases[mh]) |
| Wnt | Wnt Proteins[mh] AND Signal Transduction[mh] AND physiology[sh] AND metabolism[sh] AND (Proto-Oncogene Proteins[mh] OR Mitogen-Activated Protein Kinases[mh] OR Glycogen Synthase Kinase 3[mh]) |
